# Are the distributions of variations of circle of Willis different in different populations? – Results of an anatomical study and review of literature

**DOI:** 10.1186/1471-2377-6-22

**Published:** 2006-06-24

**Authors:** Behzad Eftekhar, Majid Dadmehr, Saeed Ansari, Mohammad Ghodsi, Bashir Nazparvar, Ebrahim Ketabchi

**Affiliations:** 1 Sina Trauma and Surgery Research Center and Department of Neurosurgery, Sina Hospital, Tehran University, Tehran, Iran

## Abstract

**Background:**

Previous studies have proposed correlation between variants of the cerebral arterial circle (also known as circle of Willis) and some cerebrovascular diseases. Differences in the incidence of these diseases in different populations have also been investigated. The study of variations in the anatomy of the cerebral arterial circle may partially explain differences in the incidence of some of the cerebrovascular diseases in different ethnic or racial groups.

While many studies have investigated the variations in the anatomy of each segment of the cerebral arterial circle, few have addressed the variants of the cerebral arterial circle as a whole. Similarly, the frequency of occurrence of such variants in different ethnic or racial groups has not been compared.

**Methods:**

102 brains of recently deceased Iranian males were dissected, in order to observe variations in the anatomy of the cerebral arterial circle. The dissection process was recorded on film and digitized. One resized picture from each dissection, showing complete circle has been made available online. The variations of the circle as whole and segmental variations were compared with previous studies.

**Results:**

On the whole, the frequencies of the different variants of the entire cerebral arterial circle and segmental variations were comparable with previous studies.

More specifically variants with uni- and bilateral hypoplasia of posterior communicating arteries were the most common in our study, similar to the previous works. No hypoplasia of the precommunicating part of the left anterior cerebral artery (A1), aplasia of A1 or the precommunicating part of the posterior cerebral artery (P1) was seen. In 3% both right and left posterior communcating arteries were absent.

**Conclusion:**

The anatomical variations found in the cerebral arterial circle of the Iranian males in the current study were not significantly different to those of more diverse populations reported in the literature. While taking into account potential confounding factors, the authors conclude that based on available studies, there is no evidence suggesting that the distributions of the variations of cerebral arterial circle differ in different populations.

## Background

The cerebral arterial circle (CAC, also known as circle of Willis, Circulus Arteriosus Cerebri) was described by Thomas Willis in 1664, but in majority of cases there are some variations from the original definition.

Previous studies have proposed correlation between variants of the CAC and some cerebrovascular diseases [1-4] and also differences in incidence of these diseases in different populations [5-10]. It seems that different distribution of variations of the CAC may partially explain the different incidence of some of the cerebrovascular diseases in different ethnic or racial groups.

The CAC is the main route for collateral blood flow in severe occlusive diseases of the internal carotid artery. Those patients with variants of the circle with efficient collateral circulation have a lower risk of transient ischemic attack (TIA) and stroke than that of patients without such collaterals[1,2]. The incidence of ischemic stroke is different among different populations, especially blacks and Hispanics compared with whites [10].

There are studies of racial variations in location and risk of intracerebral hemorrhage which can not be explained completely by known risk factors [5].

Other studies have found a correlation between cerebral aneurysms and certain variations of the CAC [3]. Finally, although still controversial [11-13], there are studies that demonstrate the existence of ethnic differences in the incidence of intracranial aneurysms [6-9].

Whether the distribution of variations in different populations is similar or not may help us in clarifying the potential role of variations of the CAC in some of the cerebrovascular diseases.

Many previous studies have investigated the variations of each segment of the CAC [4,14-21] but few [18,22-25] have addressed the variants of the CAC as a whole. Thus, it is not clear whether the different varieties of the CAC occur in similar frequencies in different ethnic or racial populations.

In order to clarify the above questions, the authors reviewed many series. Four studies were identified, which presented the variants of the entire CAC in detail (Riggs and Rupp [18], El Khamlichi [22], Fisher [23] and Lazorthes et al [25]). As there was no published anatomic series from Iran, the home country of the authors, it was considered worthwhile to undertake an autopsy study in an Iranian population. The aim of the study was to determine the variants of the CAC present in this population subset and to compare the frequencies of the different variants with previous autopsy studies.

## Methods

The present research was carried out on 102 recently deceased Iranian subjects of male gender alone, in order to control gender as one possible confounding factor. The subjects had died of natural or traumatic causes and were candidates of autopsy for medico-legal reasons. The Medico-legal office and Medical Ethics Committee of the Tehran University of Medical Sciences approved this study.

Those cases with remarkable alterations of the brain arteries or evidencing gross pathological lesions of the brain were excluded. The later category included crushed injuries, macroscopically-identified cortical tumors, severe hemorrhages or infections.

In the majority of cases, dissection around the circle was kept to a minimum.

The dissection process was filmed and digitized, so as to be readily available for further studies. Some of the earlier images were produced by digitizing analogue films taken with a Sony Handycam, but later photographs were taken with an Olympus 4.0 Megapixels digital camera. In order to avoid errors due to different angles of view, the images were taken almost perpendicular to the plane of the circles.

The following vessels forming part of the CAC were considered in the study: the anterior communicating artery (AcoA), the precommunicating part of the anterior cerebral arteries (A1), the precommunicating part of the posterior cerebral arteries (P1) and the posterior communicating arteries (PcoA).

**Figure 2:**
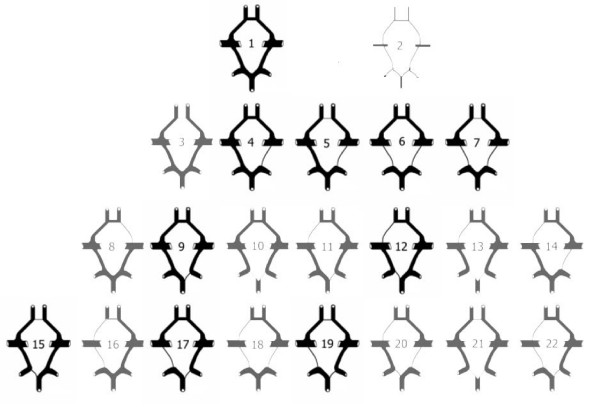
Lazorthes et al [25] classification of the cerebral arterial circle as a whole.

The authors utilized two means of comparison with previous autopsy studies. Firstly, the incomplete circles were excluded (10 cases with at least one aplastic segment – Table 1) and compared with the whole circles using Lazorthes et al [25] classification (figure 2). Secondly, the variations of each segment were compared with previous studies, using the whole dataset.

In comparison to Lazorthes et al [25], Riggs and Rupp [18] and El Khamlichi et al [22]; Fisher's [23] classification of the variations of the entire CAC was side-oriented (i.e. it was considered relevant whether the modality was on the left or the right side). As the authors of the current study used the classification of three other authors, it was necessary to reclassify Fisher's findings. On the other hand, because there were modalities that were not defined in the original classification of the Lazorthes et al [25], these modalities were classified as "others" in this study.

In order to be consistent with previous works, hypoplastic vessels were defined to be those with external diameters less than one millimeter (1 mm).

Using computer software Osiris, downloadable for free from world wide web [26], The external diameter of the vessels was measured according to the metric gauges placed along the plane of the vessels. These measurements were done on multiple images of each circle. Only one (or in one case two) resized picture from each circle, showing complete circle has been made available online [27].

**Figure 3:**
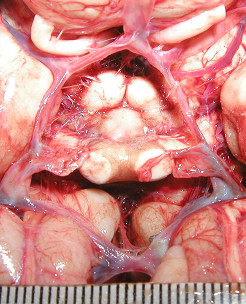
This image shows a normal cerebral arterial circle. The metric gauge placed on the plane of the vessels is shown in the bottom of the image. This meter has been used for the measurement of the diameters.

In this study's classification, segments without hypoplasia were called normal segments. (figure 3) Type 1 – all segments (AcoA, A1s, PcoAs, P1s) are normal (figure 3); Type 2 – all segments are hypoplastic; Type 3 – hypoplastic AcoA; Type 4 – hypoplastic PcoA; Type 5 – hypoplastic PcoA and AcoA; Type 6 – Bilateral hypoplastic PcoAs; Type 7 – Bilateral hypoplastic PcoAs and hypoplastic AcoA; Type 8 – hypoplastic A1; Type 9 – hypoplastic P1; Type 10 – Bilateral hypoplastic P1s; Type 11 – hypoplastic P1 and contralateral A1; Type 12 – hypoplastic P1 and ipsilateral A1; Type 13 – Bilateral hypoplastic P1s and A1; Type 14 – hypoplastic A1 and contralateral PcoA; Type 15 – hypoplastic AcoA and P1; Type 16 – hypoplastic PcoA, ipsilateral A1 and AcoA; Type 17 – hypoplastic PcoA and contralateral P1; Type 18 – Bilateral hypoplastic PcoAs and A1; Type 19 – hypoplastic PcoA, AcoA and contralateral P1; Type 20 – hypoplastic P1, contralateral PcoA and ipsilateral A1; Type 21 – Bilateral hypoplastic P1s and AcoA; Type 22 – hypoplastic PcoA, ipsilateral A1 and contralateral P1.

Since the above classification resulted in many cells with zero values, the use of common statistical methods for comparisons was not appropriate.

As several vessels were thrombosed and many were collapsed due to extravasations, measurements of the external diameters sometimes had to be estimated. In the case of fenestration of the AcoA, the diameter of the whole fenestrated vessel was measured.

In order to study the interobserver variability in the measurements, we performed paired T-test between first and second measurements done on thirteen cases.

Segmental variations were also studied and compared with similar studies in the past.

## Results

102 fresh cadavers of Iranian male subjects aged between 15 and 75 years (mean 38 ± 15 years) were dissected.

Paired T-test showed no significant statistical difference between first and second measurements done on 13 cases.

The frequencies of different types of the CAC as a whole (92 complete circles) are shown in Table 1 in comparison with the previous works of Riggs and Rupp [18], El Khamlichi [22], Fisher [23] and Lazorthes et al [25].

**Figure 4:**
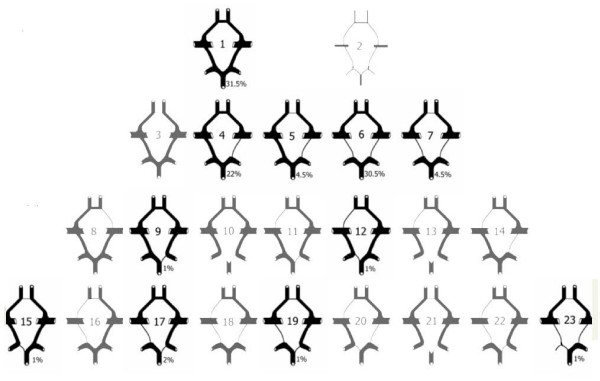
We have found 11 variants same as defined by Lazorthes et al [25]. One variant (Type 23) was not already defined.

As can be seen in figure 4, we have found 11 variants the same as those defined by Lazorthes et al [25]. One variant (Type 23) was not already defined.

The diameters of 26% of P1s on the right side and 28% on the left side were smaller than the PcoAs on the same side.

Frequencies of segmental variations using all 102 cerebral arterial circles are shown in the Table 2. Some of the previous studies have also been shown for the sake of comparison.

No hypoplasia of left A1, aplasia of A1 or P1 segments was seen. Hypoplasia of right PcoA in 16 cases (16%) and left PcoA in 11 cases (11%) were seen. In 34 cases (33%) both right and left PcoAs were hypoplastic. Aplasia of AcoA in 1 case (1%), right PcoA in 4 cases (4%) and left PcoA in 3 case (3%) was seen. In 3 cases (3%) both right and left PcoAs were absent. In those with no anastomosis between the carotid and vertebrobasilar systems, we could not find any primitive trigeminal artery, but we did not investigate the presence of other primitive caroticobasilar anastomoses.

Although this was not our primary objective, we found one aneurysm of the PcoA-P1 junction.

## Discussion

In this study the anatomical variants of the entire CAC were classified and compared with results of four previous similar studies. The segmental variations were also compared with previous published data.

Before discussing the main findings and comparisons, we would like to address the main limitations of the study.

### Limitations of the study

There are some limitations which apply generally to all such similar studies. One of them is potential minor changes in the diameter of the vessels during time. In other words, the so-called hypoplastic vessels may change to vessels with diameters larger than 1 mm during time. What we have studied is a snapshot of the circle with arbitrary definition of one millimeter as the criterion for differentiation of hypoplastic segments.

Fisher [23] and many others believed that when an artery carries collateral flow to a small territory of the brain, 1 mm diameter may be adequate. Some authors have criticized this threshold and suggested that the term hypoplasia be reserved for those vessels that cannot supply collateral flow [28], but a threshold of 1 mm has also been used by recent authors [29].

We can never be sure that our postmortem measurements are similar to those in real life. Some technical considerations may minimize the potential sources of error.

This problem may raise some concerns about reliability of our results. Our definition of hypoplasia is based on our measurements done on the images of the vessels using computer software. The measured diameters of collapsed vessels may be less precise than or different from those measured after removal of the circles and compressing them between the glass plates, formalin fixation or using injection techniques. Since other sources for measurement were not available (e.g. cerebral angiograms during lifetime) and we could not find any published data that had used such a method of measurement in medicine, we could not comment on the extent of errors. On the other hand, we have not used the absolute values of the measurements and only used them for classifications. This may minimize the extent of error and make the method acceptable for this purpose. Utilization of advanced imaging technology like MR or CT angiograms and related computer software can provide us with more precise measurements. We think that classifying the segments according to hypoplastic and normal does not seem to be realistic. Future studies should look at the circle as a dynamic structure and use classifications based on absolute values of the luminal diameters and flow of the segments. Several potential confounding factors should be considered for analysis of the results and comparisons.

### Differences in the selected study populations

We selected our cases from autopsies done on subjects in whom the apparent cause of death was either "natural" or "traumatic". This is similar to El Khamlichi et al [22] who had chosen their cases from medico-legal autopsies. Riggs and Rupp[18] had studied adults presenting with clinical manifestations of neural dysfunction. Fisher [23] had studied 414 unselected cases. Lazorthes et al [25] had not clarified their method of case selection precisely, therefore, could not be compared with ours. Differences in the selected study populations should be considered when comparing the results.

### Role of gender and age

We restricted our cases to male subjects. El Khamlichi et al [22] had studied 31 female and 69 male cases. Other studies [18,23,25] had not mentioned the sex distribution of their cases. Macchi et al [30] in his Magnetic Resonance angiography (MRA) study in 100 healthy subjects (50 men and 50 women) found no statistically significant difference between the frequency of variations between two sexes. In another MRA study [3], the authors could find sex-linked differences in anatomical variations of the CAC and could find statistically significant correlation between sex-linked differences and aneurysm distribution. Their classification has been different to the current study and Macchi et al [30]. Gender may be one of the potential sources of differences but we cannot comment on its role considering the available data.

Riggs and Rupp [18], Fisher [23] and Lazorthes et al [25] had not mentioned the age range of their cases. The age range of subjects in El Khamlichi et al [22] study was between 15 and 80 years. Due to lack of sufficient data, we cannot comment on the role of age as a potential source of difference.

### Role of race

Race may be one of the factors that may account for the differences in the distribution of the variations. Our study population was Iranian. We had excluded other races from our study, in order to control the potential confounding factor. None of the previous studies had discussed the racial homogeneity among their subjects.

### Different sets of criteria

Even though we have tried to use similar classifications, there are still some uncertainties about similarity of definitions and methods of measurement. The definition of hypoplasia has not been quantitatively defined in works of Riggs and Rupp [18] and of Lazorthes et al [25].

We addressed the issue of interobserver variability by doing a limited study on repeated measurements. Although the study showed that there were no significant statistical differences between repeated measurements, there were still a few controversial cases. These cases were classified according to the consensus among the authors.

### Comparison of the variants of the entire cerebral arterial circle

Putting aside the internal carotid segments of the CAC and classifying the segments into hypoplastic or not, we can theoretically have 64 variants of the entire circle, irrespective of the side. We have seen less than 31 variants in our and previous studies.

As is seen in Table 1, Type 6 (both PcoAs hypoplastic) variants were the most common in Lazorthes et al [25], Riggs and Rupp [18] and El Khamlichi et al [22] works. In the study done by Fisher and the present study this variant has been the second most common. More than 50% of the cases in four studies were Type 1 (complete circle with no hypoplasia), Type 4 (one hypoplastic PcoA), Type 6 (both hypoplastic PcoAs) and Type 7 (hypoplastic PcoAs and hypoplastic AcoA). Types 13 (hypoplastic P1s and A1) and 14 (hypoplastic A1 and contralateral PcoA) were found in less than 1 percent of all studies.

Although Type 3 (hypoplastic AcoA) was seen in 10% of Riggs and Rupp [18] cases and 11% of El Khamlichi et al [22] cases, it was seen in less than 5% in Lazorthes et al [25] study and 3% in Fisher's study [23]. We did not have such a variant in our study.

Type 17 (hypoplastic PcoA and contralateral P1) was seen in less than 3% of all studies except Fisher's. Fisher saw Type 17 in 11% of cases [23].

There are some similarities and differences between previous studies and ours. Considering the above- mentioned potential reasons for differences, we could not reach the conclusion that the distributions of variations of the entire CAC are different among different populations. As mentioned previously, that there were many cells with zero values, therefore, we can not support our findings with common statistical methods.

The classification used by Lazorthes et al [25] and this study is complicated. Many other simpler and more precise anatomical and functional classifications of the variants of the entire CAC might have been used for such a study. We searched the literature and found two comparable studies with such an approach. As they have used MRA for their research, they could not be compared with our study, but we compared them with each other. Since their study populations were from two different ethnic groups, we thought that their comparison might be helpful in answering our main question. Horikoshi et al [3] have classified the variations of the CAC simply into Type A, in which there was no visualization of a unilateral A1 segment, and Type P, in which there was a fetal type of posterior cerebral artery that was continuously delineated from the internal carotid artery (ICA) through the posterior communicating artery. All other variations in the CAC were defined as Type O (ordinary type of variations). They have made the results of their classification available for 440 patients without aneurysms. Macchi et al [30] in his morphologic study of 100 human healthy subjects have provided the frequency of variants similar to our study. Although both Horikoshi et al [3] and Macchi et al [30] did not make any clear comment about ethnic homogeneity of their cases, from the study environment, we could rationally assume racial differences among study populations. We reclassified the data of Macchi et al [30] an compared it with Horikoshi et al [3]. There was no significant statistical difference between distributions of variations.

Based on the above mentioned comparisons and classifications, we could not conclude that the distributions of variations of the entire CAC are different in different populations. Obviously this conclusion might be untrue using other classifications.

### Comparison of the segmental variations

As is seen in Table 2, the most common variation has been PcoA hypoplasia in all four studies.

**Figure 5:**
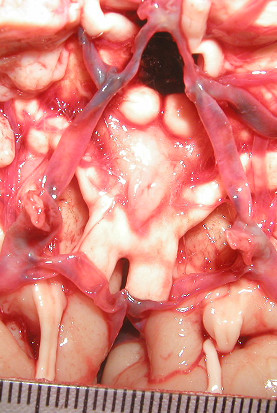
The fetal form of the posterior cerebral artery (P1 smaller than PcoA on the same side).

The fetal form of the posterior cerebral artery (P1 smaller than PcoA on the same side – figure 5) was seen in 26% of cases on the right side and 28% on the left.

In this study, on average 27% of the P1s were smaller than the PcoAs. This pattern, called fetal, is reported in the literature with percentages ranging from 10% to 40% and seems to be due to incompleteness of PcoA involution and full development of the posterior cerebral arteries [16].

In this study, aplasia of PcoA was found to be 4% on right, 3% on left and 3% on both sides. Fawcett et al [17] in their series of 350 dissections in males have reported absence of right PcoA in 2.2%, left PcoA in 0.85% and absence of both PcoAs never observed in males. In Windle's [21] study, right PcoA was absent in 4.5%, left PcoA was absent in 6.5% of cases and both PcoAs were absent in 1.5% of cases. He did not mention the gender of his patients. Our study is in agreement with Windle's [21], i.e. absent PcoAs occurred more on the left side, but interestingly, the frequency of absence of both PcoAs is higher than all the previous studies. In this group we could not find any primitive trigeminal artery, but we did not investigate the presence of other primitive caroticobasilar anastomoses.

We found one case of AcoA aplasia (1%). Piganiol et al [31] observed 14 examples (0.5%) of absent AcoA out of 2469 cases gathered from the literature.

The frequencies of the segmental variations of the CAC other than bilateral absence of PcoAs were within the range of previous studies.

Variation of the CAC has been the subject of many theories. Lazorthes et al [25] suggested that the variation of the segment calibers was due to the amplitude of the neck movements in the later years of life.

Hillen [24], in his analysis of 100 circles of Willis in adults, indicated that variation of the circle is based on a delicate hemodynamic "tuning" of all segments and that the relations between diameters of segments exists without reference to flow changes in the afferent arteries during movement of the neck.

According to Stehbens [32], topographical modification of the circle occurs during postnatal development of the brain and with arteriectasis, atherosclerosis, and secondary modification of collateral vessels in pathological occlusive disease.

Van Overbeeke and colleagues [19], examining the posterior segment of 53 complete circles of Willis on fetal and neonatal brains, concluded that variations of this segment were the result of developmental modification, especially with increased functional demands in connection with the rapid growth of the occipital lobes.

Milenkovic et al [33] based on previous works and his observations postulated that genetic factors are probably responsible not only for the development of the circle but also for its caliber. The difference in arterial calibers of the CAC during life could be the results of hemodynamic stress or compression of the afferent vessels.

Vasovic et al [34] believe that the arterial variations and abnormalities have fetal characteristics and that they are the result of a genetically established pattern. They suggest that arterial variations and abnormalities could also preserve their relationships because of constant interaction between primitive arterial remnants and brain arteries in postnatal life.

From the evolutionary standpoint, it is noteworthy that variations of the cerebral arteries seem to be equally common in humans as well as animals [35]. Although the present study should be interpreted cautiously, considering our results, we cannot say that the responsible factors for variations (either genetic or acquired) are different in different populations or act differently.

The online availability of some of the digital images may be considered an advantage of this study and helps repetition of some of the measurements and comparisons. Resizing of the images may have decreased the quality of some images. A few of the images are not of good quality, but since they could be classified, we finally decided to include them.

As discussed before, the results should be considered cautiously and need further verifications. Advanced imaging techniques can help us significantly in this regard.

## Conclusion

In this study, we presented our experience and reviewed many other series, trying to figure out whether the distribution of the variations of the CAC is similar among different populations or not.

Considering potential confounding factors, we conclude that we have no evidence that the distributions of the variations of CAC differ in different populations.

The results should be interpreted cautiously. In order to verify these findings, further studies are needed. We recommend using new imaging modalities and classifications of variants of the entire CAC based on quantitative measurements of the luminal diameters and flow of the segments.

## Competing interests

The author(s) declare that they have no competing interests.

## Authors' contributions

BE carried out the data extraction, performed the analysis and drafted the manuscript. MD and SA carried out the data extraction and participated in analysis. MG, BN and EK supervised the study and participated in its coordination. The figures were drawn by MD. All authors read and approved the final manuscript.

## Pre-publication history

The pre-publication history for this paper can be accessed here:



## Figures and Tables

**Figure 1:**
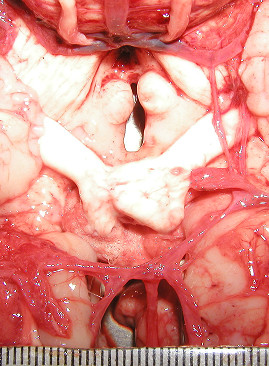
This image is an example of incomplete cerebral arterial circle with aplastic left PcoA.

**Table 1:** Our results in comparison with the previous works of Riggs and Rupp [18], El Khamlichi [22], Fisher [23] and Lazorthes et al [25].

Types	Riggs and Rupp [18]	El Khamlichi et al [22]	Fisher [23]	Lazorthes et al [25]	This study
1	192	18	20	29	29
2	54	0	0	10	0
3	91	11	6	9	0
4	88	14	24	28	20
5	41	6	12	10	4
6	126	24	131	44	28
7	67	10	58	34	4
8	38	2	0	3	0
9	47	3	4	5	1
10	33	1	16	6	0
11	2	0	10	0	0
12	20	1	1	3	1
13	5	0	3	1	0
14	7	0	1	1	0
15	35	4	0	4	1
16	16	3	2	2	0
17	26	0	46	3	2
18	58	0	21	6	0
19	17	1	28	1	1
20	10	1	5	0	0
21	13	0	10	1	0
22	3	0	8	0	0
Others	5	1	8	0	1

**Table 2:** Frequencies of segmental variations using all 102 circles of Willis in comparison with the previous studies.

Variations	No	AcoA Aplasia	AcoA Hypoplasia	AcA Aplasia	AcA Hypoplasia	PcoA Aplasia		PcoA Hypoplasia		P1 Hypoplasia		P1 Aplasia
							
References						Uni	Bi	Uni	Bi	Uni	Bi	
Alpers [14]	350	2%	3%	0	2%	0.6%	0	13.5%	9%	11%^1^	3.71%^1^	
Fawcett [17]	700	0.14%	-	-	-	3.2%	0.4%	21.5%	0.7%	42%^1^	0.14%^1^	-
Riggs & Rupp [18]	994		27%		16%			21%	30%	16%	6%	-
Windle [21]	200	1.5%	-	-	-	11%	1.5%	21.5%	3.5%	10%^1^	2%^1^	
El khamlichi Et al [22]	100		32%		4%			25%	35%	14%	1%	-
Fisher [23]	414		29%		13%			32%	49%	22%	7%	
Lazorthes et al [25]	200		29.5%		7.5%			29.5%	40%	7.5%	4.5%	-
Puchades [36]	62	3.2%	6.4%	0.8%	6.4%	2.4%^2^		51.6%		11.3%	11.3%^1^	2.4%
Present study	102	1%	11%	-	1%	7%	3%	27%	33%	7%	-	

^1^Fetal form of the posterior cerebral artery (P1 smaller than PcoA on the same side)

^2^The study has not indicated whether it has included bilateral hypoplasia or not.
